# Regulation of High-Altitude Hypoxia on the Transcription of CYP450 and UGT1A1 Mediated by PXR and CAR

**DOI:** 10.3389/fphar.2020.574176

**Published:** 2020-09-17

**Authors:** Ya-bin Duan, Jun-bo Zhu, Jian-xin Yang, Gui-qin Liu, Xue Bai, Ning Qu, Xue-jun Wang, Xiang-yang Li

**Affiliations:** ^1^ Research Center for High Altitude Medicine, Qinghai University Medical College, Xining, China; ^2^ State Key Laboratory of Plateau Ecology and Agriculture, Qinghai University, Xining, China; ^3^ College of Eco-Environmental Engineering, Qinghai University, Xining, China; ^4^ Department of Anesthesiology, Qinghai Hospital of Traditional Chinese Medicine, Xining, China; ^5^ Department of Anesthesiology, Red Cross Hospital of Qinghai, Xining, China

**Keywords:** high-altitude hypoxia, cytochrome P450, UDP-glucuronosyltransferase 1A1, pregnane X receptor, constitutive androstane receptor

## Abstract

Little is known about what roles the pregnane X receptor (PXR) and constitutive androstane receptor (CAR) play in drug metabolism in high-altitude hypoxia. Likewise, the potential interaction of nuclear receptors and drug metabolism enzymes during drug metabolism of high-altitude hypoxia is not fully understood. In this work, we investigated the effects of high-altitude hypoxia on transcriptional regulation of cytochrome P450 (CYP450) and UDP-glucuronosyltransferase 1A1 (UGT1A1) genes mediated by PXR and CAR proteins. The protein and mRNA expressions of CYP450, UGT1A1, PXR, and CAR were determined by enzyme-linked immunosorbent assay and qPCR in rats and HepG2 cell lines under hypoxia. Hypoxia potently inhibited the CYP450 isoforms, UGT1A1, PXR, and CAR protein and mRNA expression. To clarify whether PXR and CAR regulate various genes involved in drug metabolism of high-altitude hypoxia, we investigated the expression of CYP1A2, CYP2C9, CYP2E1, CYP3A4, and UGT1A1 using a dual-luciferase reporter assay after treatment with Ketoconazole (KCZ) and Retinoic acid (RA), or silenced PXR and CAR gene expression. In HepG2 cells, hypoxia, KCZ, and RA inhibited CYP450 isoforms and UGT1A1 expression. Activation of PXR and CAR in cells treated with 6-(4-chlorophenyl)-imidazo (2,1-b) thiazole-5-carbaldehyde (CITCO) and rifampicin (Rif) resulted in the enhancement of CYP450 isoforms, UGT1A1, PXR, and CAR. In contrast, this effect was not observed under hypoxia. Taken together, our results suggest that hypoxia inhibits CYP1A2, CYP2C9, CYP2E1, CYP3A4, and UGT1A1 expression *via* the PXR and CAR regulatory pathway.

## Introduction

The high-altitude environment is characterized by increased solar radiation, decreased ambient oxygen tension, extreme diurnal ranges in temperature, arid climate, and poor soil quality; of these, hypoxia is the main factor with the potential to affect human life and activity (Eide and Asplund, 2012). The atmospheric pressure and available oxygen decrease with increasing altitude. There are four levels of high-altitude hypoxia: light hypoxia (1,500–2,500 m), moderate hypoxia (2,500–4,500 m), severe hypoxia (4,500–5,500 m), and extremely severe hypoxia (5,500–8800 m). There are many places on the planet above 3,000 m that humans live in conditions of hypobaric hypoxia, which is known to induce physiological changes in most people (Eide and Asplund, 2012). There are three main regions that host human populations over 4,000 m: the Tibetan plateau and Himalayan valleys, the South American Andes, and the Ethiopian Highlands (Gilbert-Kawai et al., 2014; West, 2017). Of these, the Qinghai-Tibetan plateau is the largest and highest in the world, with almost 9 million inhabitants living in long-term high-altitude hypoxia. In recent years, more people have moved to the plateau regions due to social and economic development (Zhou et al., 2018). Hypoxia-induced fluctuations in organism function, metabolism, and structure underly the pathological basis of disease at high altitude (Zhou et al., 2018). High-altitude hypoxia has negative effects on the cardiovascular system, nervous system, and metabolism, which contribute to potential changes in drug metabolism (Mairbaurl, 1994). The altitude and duration of exposure to hypoxia also play a key role in drug metabolism.

Numerous studies have focused on the mechanisms underlying high-altitude pulmonary edema (HAPE) and high-altitude cerebral edema (HACE), prevention of altitude sickness, and adaptation to hypoxia (Dehnert and Batsch, 2017; He et al., 2017; Jin, 2017). More recently, changes in drug metabolism under high-altitude hypoxia have drawn increasing scientific attention. High altitude induces physiological and pathological changes resulting in altered drug absorption, distribution, metabolism, and excretion (Anjana et al., 2012). For example, the clearance (CL) and volume of distribution (Vd) of diltiazem were significantly decreased under hypoxia. Hypoxia also altered the disposition of propranolol, significantly decreasing the area under the curve (AUC), mean residence time (MRT), half-life (T_1/2_), and peak concentration (C_max_) (Zhou et al., 2018). The pharmacokinetic characteristics of drugs can be affected by changes in drug metabolism enzymes induced by hypoxia.

Cytochrome P450 enzymes are highly versatile components of an oxidase system encoded by a large gene family. CYP1A, CYP2B, CYP2C, CYP2E, and CYP3A are the main subtypes; more than 90% of the drugs are metabolized by these enzymes (Shah et al., 2016). CYP3A4 is the most important drug-metabolizing enzyme, converting 50% of drugs to their metabolites (Zhang et al., 2016; Shah et al., 2016; Li M. M. et al., 2017). Fradette et al. found that acute hypoxia decreased the expression of CYP1A1, CYP1A2, CYP2B4, CYP2C5, and CYP2C16 (Fradette et al., 2003; Fradette and Du, 2004; Fradette et al., 2007). In our previous study, we found that hypoxia depresses CYP1A2, CYP2C11, CYP3A1, and CYP2C22 (Li et al., 2012b; Li et al., 2014a; Li et al., 2014b).

Major cytochrome P450 and drug metabolism enzymes are regulated by nuclear receptors, namely the pregnane X receptor and the constitutive androstane receptor. The pregnane X receptor and constitutive androstane receptor pathways are highly important for CYP450 transcriptional regulation. Many studies have shown that the transcription of CYP3A, CYP2B, and CYP1A is mediated by PXR and CAR (Ratejewski et al., 2011; Park et al., 2012). Gene knockdown studies have demonstrated that PXR and CAR regulate the expression of CYP2C9, CYP2C19, and CYP2C18 (Chen et al., 2004; Hu et al., 2011). These receptors establish a cross-talk with other signaling pathways, which further complicates the mechanisms of molecular regulation mediated by PXR and CAR in drug metabolism enzymes in high-altitude hypoxia. However, the mechanisms underlying the effects of high-altitude hypoxia on CYP450 and UGT1A1 still remain unclear. To address this knowledge gap and identify mechanisms regulating the drug metabolism enzymes, we aimed to investigate the effect of high-altitude hypoxia on the regulation of CYP450 and UGT1A1 transcription mediated by PXR and CAR.

## Materials and Methods

### Reagents and Chemicals

Enzyme-linked immunosorbent assay (ELISA) kits used to evaluate rat CYP1A2 (Lot: L150924006), CYP2B1 (Lot: 201511), CYP2C11 (Lot: L150924092), CYP2C22 (Lot: 201511), CYP2D1 (Lot: L150928285), CYP2E1 (Lot: L150924112), CYP3A1 (Lot: L150924046), UGT1A1 (Lot: L150928282), PXR (Lot: L150928308), and CAR (Lot: L150928297) were purchased from USCN Life Science Inc. (Wuhan, China*)*. ELISA kits used to evaluate HepG2 CYP1A2 (Lot: L150923651), CYP2B6 (Lot: L151029739), CYP2C9 (Lot: L150923653), CYP3A4 (Lot: L151029738), UGT1A1 (Lot: L150923656), PXR (Lot: L150923657), and CAR (Lot: L151029737) were purchased from USCN Life Science Inc. (Wuhan, China*).* RNAiso Plus and PrimeScript™ RT Reagents were obtained from Takara Bio (Kyoto, Japan). Primers for real-time PCR were synthesized by Shanghai Sangon Biotech Co., Ltd. (Shanghai, China). *Escherichia coli* DH5α cells (Lot: 20160829) were obtained from Solarbio Life Science Inc. (Beijing, China). The HepG2 cell line was obtained from the Cell Bank of the Chinese Academy of Science (Shanghai, China). Lipofectamine 2000 Transfection Reagent (Lot: CT001) was purchased from R&S Biotech Co., Ltd. (Shanghai, China). Dual-Luciferase Reporter Assay System (Lot: E1910) was purchased from Promega Biotech Co., Ltd. (Madison, WI, USA). Ketoconazole (KCZ, Lot: VRTIO-AJ) and Rifampicin (Rif, Lot: ZFEPB-CE) was purchased from Tokyo Chemical Inc. (Kyoto, Japan). Retinoic acid (RA, Lot: P3VLM-BN) was purchased from TCI Shanghai Co., Ltd. (Shanghai, China). CITCO (Lot: C6240) was purchased from Sigma-Aldrich Chemical Company (St Louis, MO, USA). siRNA-PXR (Lot: siB11922163057) and siRNA-CAR (Lot: tB0005365C) were obtained from RiboBio Co., Ltd. (Shanghai, China).

### Animals and Experimental Treatments

Fifty Sprague Dawley (SD) SPF inbred strain rats (200 ± 20 g, equal number of male and female rats) were obtained from the laboratory animal center of Xi’an Jiaotong University Medical College (Certificate No.: 2012-003, Xi’an, China). All experimental procedures were performed in strict accordance with the National Institutes of Health Guide for the Care and Use of Laboratory Animals. The protocol was approved by the Animal Ethics Committee of The Qinghai University. Ten animals of the same sex were housed in each cage in separate rooms to ensure that each animal could be restrained within a single space to reduce stress. Animals were adapted for 1 week at 23 ± 2°C with a constant humidity of 55 ± 5% under a 12 h dark cycle and given *ad libitum* access to water and food pellets.

Fifty SD rats were randomly divided into five groups, each containing five male and five female rats. The plain group (P, altitude: 390 m, 23 ± 2°C, PaO_2_: 20 kPa, relative humidity: 55 ± 5%) included rats living in the city of Xi’ an in northwest China’s Shanxi Province. The acute moderate-altitude hypoxia group (MAH, altitude: 2,800 m, 23 ± 2°C, PaO_2_: 15.1 kPa, relative humidity: 55 ± 5%) and the chronic moderate-altitude hypoxia group (MCH, altitude: 2,800 m, 23 ± 2°C, PaO_2_: 15.1 kPa, relative humidity: 55 ± 5%) were comprised of healthy rats living at plain level but having undergone 24 h acute exposure and 30-day chronic exposure to moderate altitude, respectively. The rats in these two groups were transported by bus to Gonghe County, which is in the Northwestern Qinghai Province of China. The acute high-altitude hypoxia group (HAH, altitude: 4,300 m, 23 ± 2°C, PaO_2_: 12.4 kPa, relative humidity: 55 ± 5%) and the chronic high-altitude hypoxia group (HCH, altitude: 4,300 m, 23 ± 2°C, PaO_2_: 12.4 kPa, relative humidity: 55 ± 5%) were comprised of healthy rats living at plain level, but having undergone 24 h acute exposure and 30-day chronic exposure to high altitude, respectively. The rats in these two groups were transported by bus to the Huashixia Town, also in the Qinghai Province. Blood and hepatic tissue from MAH, MCH, HAH, and HCH rats were collected following 24 h acute and 30-day chronic exposure to high or moderate altitudes. The P group was examined at the Xi’an Jiaotong University Medical College. Animals were immediately anesthetized *via* enterocoelia injection with sodium pentobarbital (50 mg/kg) prior to the experimental procedures.

### Physiological and Blood Parameters

Venous blood was collected from all rats, and red blood cells (RBC), white blood cells (WBC), and hemoglobin (HGB) were determined using an XFA6100 automatic hemocyte analyzer (Perlong Medical Inc, China). Blood oxygen saturation (S_c_O_2_) was measured using a TUFFSAT oximeter (Ohmeda medicalMedical Inc, USA). Albumin (ALB) and bilirubin (BIL) were determined by an AU2700 automatic biochemistry analyzer (Olympus Inc, Japan).

### Preparation of Rat Hepatic Microsomes

Liver microsomes were prepared by differential centrifugation as previously described (Li et al., 2014; Li et al., 2014). Briefly, liver samples were thawed and weighed, and two volumes of ice-cold homogenization medium (50 mmol.L^-1^ Tris-HCl buffer at pH 7.4 containing 0.25 mol.L^-1^ sucrose) were then added. The tissue was chopped using scissors and homogenized with an automatic homogenizer at 500 rpm (IKA Labortechnik, Germany). The resultant homogenates were transferred to clean centrifuge tubes and centrifuged at 10,000 g for 30 min at 4°C using a TGL-16B Anting centrifuge (Anting Scientific Instrument Factory, China). The supernatant was collected and centrifuged at 603,680 g for 80 min at 4°C using an Optima MAX-XP ultracentrifuge (Beckman Coulter Inc, USA). The microsomal pellet was resuspended in homogenization medium. Hepatic microsomal suspensions (0.5 ml) were aliquoted into Eppendorf tubes and stored at −80°C until batch processing.

### ELISA Analysis of CYP1A2, CYP2B1, CYP2C11, CYP2C22, CYP2D1, CYP2E1, CYP3A1, UGT1A1, PXR, and CAR Protein Expression in Rats

Protein expression of CYP1A2, CYP2B1, CYP2C11, CYP2C22, CYP2D1, CYP2E1, CYP3A1, UGT1A1, PXR, and CAR was evaluated by ELISA according to the manufacturer’s protocol. Briefly, liver tissue was removed from rats, and hepatic microsomes were prepared accordingly. Standard diluents and Str-HRP-Conjugate Reagent (50 µl of each) were added to standard orifices containing combined biotin antibody. In turn, 40 µl of each sample, 10 µl of an antibody targeting the protein of interest, and 50 µl Str-HRP-Conjugate Reagent were added to the orifice. The plate was covered, sealed, and incubated at 37°C for 60 min with gentle shaking. The liquid was discarded, the plate was spin-dried, washed using an automatic plate washer, and finally patted dry. Chromogen solution A and B (50 µl of each) was added to each well, mixed gently, and samples were then incubated for 15 min at 37°C in the dark. The reaction was stopped by the addition of 50 µl stop solution, and the optical density (OD) at 450 nm was measured within 15 min.

### RNA Isolation and Real-Time PCR Analysis of CYP1A2, CYP2B1, CYP2C11, CYP2C22, CYP2D1, CYP2E1, CYP3A1, UGT1A1, PXR, and CAR mRNA Expression in Rats

Fifty to 100 mg of liver tissue was homogenized and total RNA was isolated using TRIzol reagent. The quality of the RNA solutions was assessed using a NanoDrop 2000c spectrophotometer (Thermo Fisher Scientific, USA). cDNA was synthesized according to the manufacturer’s protocol. Relative mRNA expression was analyzed using the BIO-RAD CFX Connect Real-Time PCR System (Bio-Rad Laboratories, Inc., USA). Products were amplified at 95°C for 2 min, followed by 40 cycles at 95°C for 15 s, 60°C for 20 s, 72°C for 20 s, and 60–95°C for 15 s. Fold induction values were calculated according to the equation 2^–ΔΔCt^, where ΔCt represents the difference in cycle threshold numbers between the target gene and the control gene β-actin, and ΔΔCt represents the relative change in the difference between the control and treatment groups. The primers are listed in **Table 1**.

**Table 1:** Rat primers used for real-time PCR analysis.

Gene	Oligonucleotide primer sequences (5′-3′)	
PXR	Forward	CCCACCTCAGAAGACAAAGC
	Reverse	GAACCCCAGACCCTACACAA
CAR	Forward	TACTGTGCTTCGTGCTCCTG
	Reverse	CCTGGTCTTCGGGTTCAAG
CYP1A2	Forward	CATAGCCTCAGACCCCACAT
	Reverse	ACATTAGCCACCGATTCCAC
CYP2B1	Forward	GTGGAAGAACGGATTCAGGA
	Reverse	AGCAGATGATGTTGGCTGTG
CYP2C11	Forward	GAGGACCATTGAGGACCGTA
	Reverse	GAGCACAGCCCAGGATAAAG
CYP2C22	Forward	ATGGGGATGGGAAAGAGAAC
	Reverse	TGCTGGAAAATGACACTGGA
CYP2D1	Forward	TCCGTGCTGAAGGATGAGA
	Reverse	GAGGCAGGTGAAGAAGAGGA
CYP2E1	Forward	TGGGGAAACAGGGTAATGAG
	Reverse	CAATCAGAAATGTGGGGTCA
CYP3A1	Forward	AAATGCCTCTGTTTGCCATC
	Reverse	CTTTCCCCATAATCCCCACT
UGT1A1	Forward	ATCGTGTTGACGGTGGTCTT
	Reverse	TGGGTCTTGGATTTGTGTGA
β-actin	Forward	TCACCAACTGGGACGATATG
	Reverse	GTTGGCCTTAGGGTTCAGAG

The RNA samples were of sufficient quality for qPCR analysis. Protein-free and intact RNA were indicated by purity and integrity assessment of total RNA. qPCR efficiency over all samples was calculated according to the Minimum Information for Publication of Quantitative Real-Time PCR Experiments (MIQE) guidelines. Primer specificity was confirmed by melting curve analysis. The specific amplification of target reference genes was assessed by a specific peak in melting curve analysis (**Supplementary Figures S1–S3**).

### Cell Culture and Treatment

The hepatoblastoma cell line HepG2 was maintained in 6 cm culture plates with minimal essential medium supplemented with 10% fetal bovine serum and incubated at 37°C and 5% CO_2_. HepG2 cells were plated at a density 2 × 10^5^ cells per well into six-well plates. HepG2 cells were treated with various oxygen concentrations or PXR inhibitors (Ketoconazole, KCZ, 10 μmol/L), CAR inhibitors (Retinoic acid, RA, 10 μmol/L), or the solvent control (0.1% DMSO) for 24 h in normoxia. Oxygen concentrations and treated time were as follow: 2% O_2_ for 24 h (2%-24 h), 5% O_2_ for 24 h (5%-24 h), 10% O_2_ for 24 h (10%-24 h), 5% O_2_ for 2 h (5%-2 h), 5% O_2_ for 6 h (5%-6 h), 5% O_2_ for 12 h (5%-12 h), 5% O_2_ for 24 h (5%-24 h), 5% O_2_ for 48 h (5%-48 h), 21% O_2_ for 24 h (normoxia). In the agonist experiment, HepG2 cell were treated with rifampicin (10 μmol/L, PXR agonist) and CITCO (10 μmol/L, CAR agonist) for 2, 6, 16, or 24 h in normoxia. There were three replicates in each condition. The cells were harvested and the protein and mRNA levels of CYP450, UGT1A1, PXR, and CAR were determined by ELISA and real-time PCR according to the manufacturer’s protocol. The primers used are listed in **Table 2**.

**Table 2:** HepG2 primers used for real-time PCR analysis.

Gene	Oligonucleotide primer sequences (5′-3′)	
PXR	Forward	TCATGGCTATGCTCACCGAG
	Reverse	CTGTGATGCCGAACAACTCC
CAR	Forward	TGGGGTTCCAGGTAGAGTTTT
	Reverse	CCAGGTCGGTCTGTAAGATAGG
CYP1A2	Forward	CCCAGTCTGTTCCCTTCTCG
	Reverse	TGGCTCTGGTGGACTTTTCA
CYP2B6	Forward	GGACCTCATCGACACCTACCT
	Reverse	TCACCTGTTCAATCTCCCTGTA
CYP2C9	Forward	CTGAAACCCATAGTGGTGCTG
	Reverse	GAAACGCCGGATCTCCTT
CYP3A4	Forward	CTTTTGGTCCAGTGGGATTTA
	Reverse	CGTCTTTCAAGGTGACAGGCT
UGT1A1	Forward	CAAAGGGAGGATGTGAAAGAGT
	Reverse	CAAGAAGAATACAGTGGGCAGA
β-Actin	Forward	GGCACTCTTCCAGCCTTCC
	Reverse	GAGCCGCCGATCCACAC

### Plasmid Construction

To construct CYP1A2, CYP2C9, CYP2E1, CYP3A4, and UGT1A1 plasmid vector, the promoter region were amplified, and CYP1A2, CYP2C9, CYP2E1, CYP3A4, and UGT1A1 primers were designed (**Table 3**). Amplifications were performed in a volume of 50 μl with an initial pre-denaturation step at 94°C for 3 min. This was followed by 35 cycles of 94°C for 30 s, 58°C for 30 s, and 68°C for 30 s. Amplification products were resolved by agarose gel (1%) electrophoresis and detected by ethidium bromide. Target bands were visualized under UV light and photographed with a computer-assisted camera. The PCR products for CYP1A2, CYP2C9, CYP2E1, CYP3A4, and UGT1A1 were digested and ligated into a pGL3 vector by T4 DNA ligase. The 10 μl connection products were transformed into DH5α cells, which were plated in ampicillin plates and incubated overnight at 37°C. Single colonies were picked and cultured for 12 h with amp^+^-LB liquid. The plasmids were named pGL3-CYP1A2, pGL3-CYP2C9, pGL3-CYP2E1, pGL3-CYP3A4, and pGL3-UGT1A1. Monoclonal cells were selected and cultured. Gene fragments in the recombinant clones were confirmed by sequencing (**Supplementary Figures S4–S6**) (Chang et al., 2014).

**Table 3:** Primers used for vector construction.

Gene	Oligonucleotide primer sequences (5′-3′)	
CYP1A2	nheI-Forward	ACTACGCTAGCAAACAACTTTCCTCTTCTCCCATTC
	hindIII-Reverse	TAGCTAAGCTTTTGGAAGGATCAACTCTTGGCCTTG
CYP2C9	mluI-Forward	ACTTAACGCGTGCAAAGTTTAGAGTAGTTGATCTCA
	xhoI-Reverse	ACTTACTCGAGTAGGTCCACTATATGCTCCTTCTGA
CYP2E1	nheI-Forward	ACTACGCTAGCACGTACACTGACACAGAGATATTCT
	hindIII-Reverse	TAGCTAAGCTTAGAGGGCGCGGGGCTCTGTGCTGT
CYP3A4	nheI-Forward	ACTACGCTAGCCTCCCATCAGGAATGGGTCAGG
	hindIII-Reverse	TAGCTAAGCTTGTTAAAGGAGAATGGTTATAAGATC
UGT1A1	nheI-Forward	ACTACGCTAGCTTCTGGCTGCACAATACTTGCCCCA
	hindIII-Reverse	TAGCTAAGCTTCCCCACCTCACCACCACTTCTGGAA

Amplification products were analyzed by agarose gel (1%) electrophoresis. The fragments conformed to the designed length standards of CYP1A2 (1,901 bp), CYP2C9 (2,051 bp), CYP2E1 (2,100 bp), CYP3A4 (2,001 bp), and UGT1A1 (2,051 bp) (**Supplementary Figure S7**). pGL3-CYP1A2, pGL3-CYP2C9, pGL3-CYP2E1, pGL3-CYP3A4, and pGL3-UGT1A1 vectors were successfully constructed following verification by sequencing.

### Transient Transfections of Cells

One day before transfection, cells were trypsinized and plated into 24-well plates at 37°C and 5% CO_2_. Cells were allowed to reach 60–70% confluency at the time of transfection. To assess CYP450 promoter activity following treatment with nuclear receptor inhibitors (KCZ, 10 mmol/L; RA, 10 mmol/L), nuclear receptor agonists (RIF, 10 mmol/L; CITCO, 10 mmol/L) or hypoxia, HepG2 cells were transfected with 1 μg of the pGL3-CYP450 or empty vector using Lipofectamine Plus, and the obtained cells were cultured in a 5% CO_2_ incubator at 37°C for 4–6 h (Chang et al., 2014; Kanno et al., 2016).

### Luciferase Reporter Assays

HepG2 cell were transfected with transfection reagent according to the manufacturer’s instructions. After transfection, HepG2 were treated with hypoxia or normoxia and solvent (0.1% DMSO) or PXR/CAR inhibitors. Subsequently, cell lysates were assayed for firefly activities normalized against the activity of cotransfected renilla luciferase using a dual-luciferase kit (Promega, USA).

### PXR and CAR Gene Silencing

The siRNA-PXR and siRNA-CAR was designed and synthesized by RiboBio (Shanghai, China). HepG2 cells were transfected with 0.5 μg of siRNA-PXR or siRNA-CAR using Lipofectamine Plus, according to the manufacturer’s protocol. Following transfection with the siRNA-PXR and siRNA-CAR vectors, cells were incubated for 24 h and harvested to extract RNA (**Supplementary Figure S8**). The mRNA expression of CYP1A2, CYP2C9, CYP2E1, CYP3A4, and UGT1A1 was detected by qPCR. The primers used are listed in **Table 4**.

**Table 4:** Primers for CYP1A2, CYP2C9, CYP2E1, CYP3A4, and UGT1A1.

Gene	Oligonucleotide primer sequences (5′-3′)	
CYP1A2	Forward	CCCAGTCTGTTCCCTTCTCG
	Reverse	TGGCTCTGGTGGACTTTTCA
CYP2C9	Forward	CTGAAACCCATAGTGGTGCTG
	Reverse	GAAACGCCGGATCTCCTT
CYP2E1	Forward	CCCATCATCGGGAACCTC
	Reverse	TAGCCGTGCATCACCACC
CYP3A4	Forward	CTTTTGGTCCAGTGGGATTTA
	Reverse	CGTCTTTCAAGGTGACAGGCT
UGT1A1	Forward	CAAAGGGAGGATGTGAAAGAGT
	Reverse	CAAGAAGAATACAGTGGGCAGA
β-Actin	Forward	GGCACTCTTCCAGCCTTCC
	Reverse	GAGCCGCCGATCCACAC

### Statistical Analysis

All quantitative data are expressed as mean ± standard error of the mean (SEM). The data were analyzed using one-way analysis of variance (ANOVA) in the statistical package for social sciences version 23.0 (IBM, Armonk, NY, USA). Differences between the means of the two groups were compared using Least Significant Difference (LSD) *post hoc* tests. The results were considered statistically significant when *P* < 0.05.

## Results

### Physiologic and Blood Parameters

**Figure 1:**
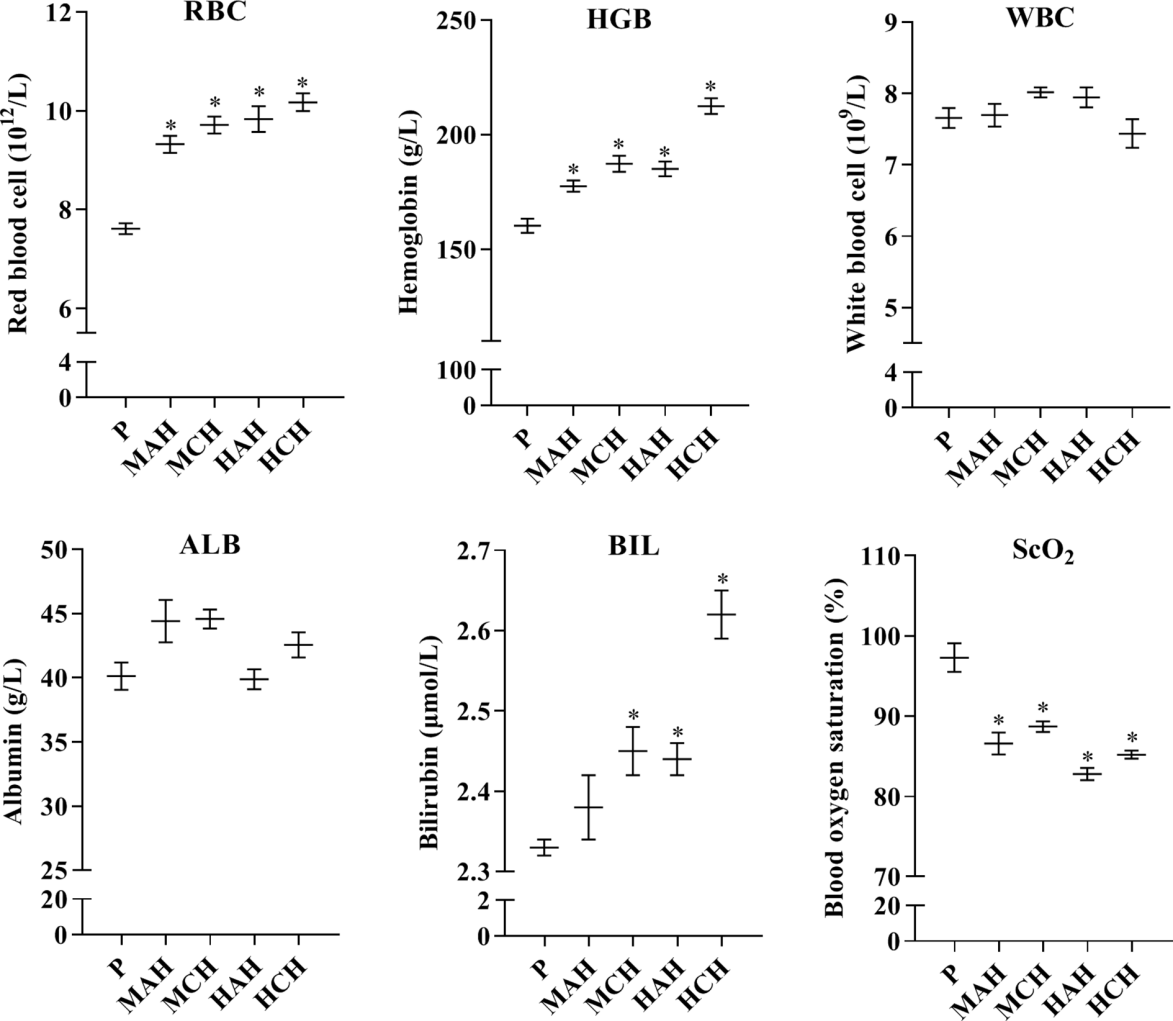
Changes in red blood cells, white blood cells, hemoglobin, blood oxygen saturation, albumin, and bilirubin from rats after exposure hypoxia. P: plain group (altitude: 390 m, PaO_2_: 20 kPa), MAH: acute moderate-altitude hypoxia group (altitude: 2,800 m, PaO_2_: 15.1 kPa; Rats were exposed to moderate altitude for 24 h), MCH: chronic moderate-altitude hypoxia group (altitude: 2,800 m, PaO_2_: 15.1 kPa; Rats were exposed to moderate altitude for 30 days), HAH: acute high-altitude hypoxia group (altitude: 4,300 m, PaO_2_: 12.4 kPa; Rats were exposed to high altitude for 24 h), HCH: chronic high-altitude hypoxia group (altitude: 4,300 m, PaO_2_: 12.4 kPa; Rats were exposed to high altitude for 30 days). The data were analyzed using a one-way analysis of variance (ANOVA), and the differences between the means of two groups were compared using LSD tests. ^*^ indicates the significance of experimental groups, compared with that of the control. Values are expressed as Mean ± SEM (n = 10). Levels are considered significant at ^*^ *p <* 0.05.

To assess physiological changes in rats exposed to high-altitude hypoxia and to confirm the relationship between these changes and drug metabolism, physiological indexes and blood cells were examined. Red blood cells were significantly increased by 22.5, 27.6, 29.2, and 33.6% in the MAH, MCH, HAH, and HCH groups, respectively, compared with the P group. Hemoglobin levels were increased by 10.8, 16.9, 15.5, and 32.6% in the MAH, MCH, HAH, and HCH groups, respectively, compared with the P group. However, there was no significant difference in WBC and ALB between groups. S_C_O_2_ values were decreased by 11.0, 8.6, 14.9, and 12.4% in the MAH, MCH, HAH, and HCH groups, respectively, compared with the P group. The BIL values were significantly increased by 5.2, 4.7, and 12.4% in the MCH, HAH, and HCH groups, respectively, compared with the P group (**Figure 1**).

### Protein Expression of CYP1A2, CYP2B1, CYP2C11, CYP2C22, CYP2D1, CYP2E1, CYP3A1, UGT1A1, PXR, and CAR in Rats

**Figure 2:**
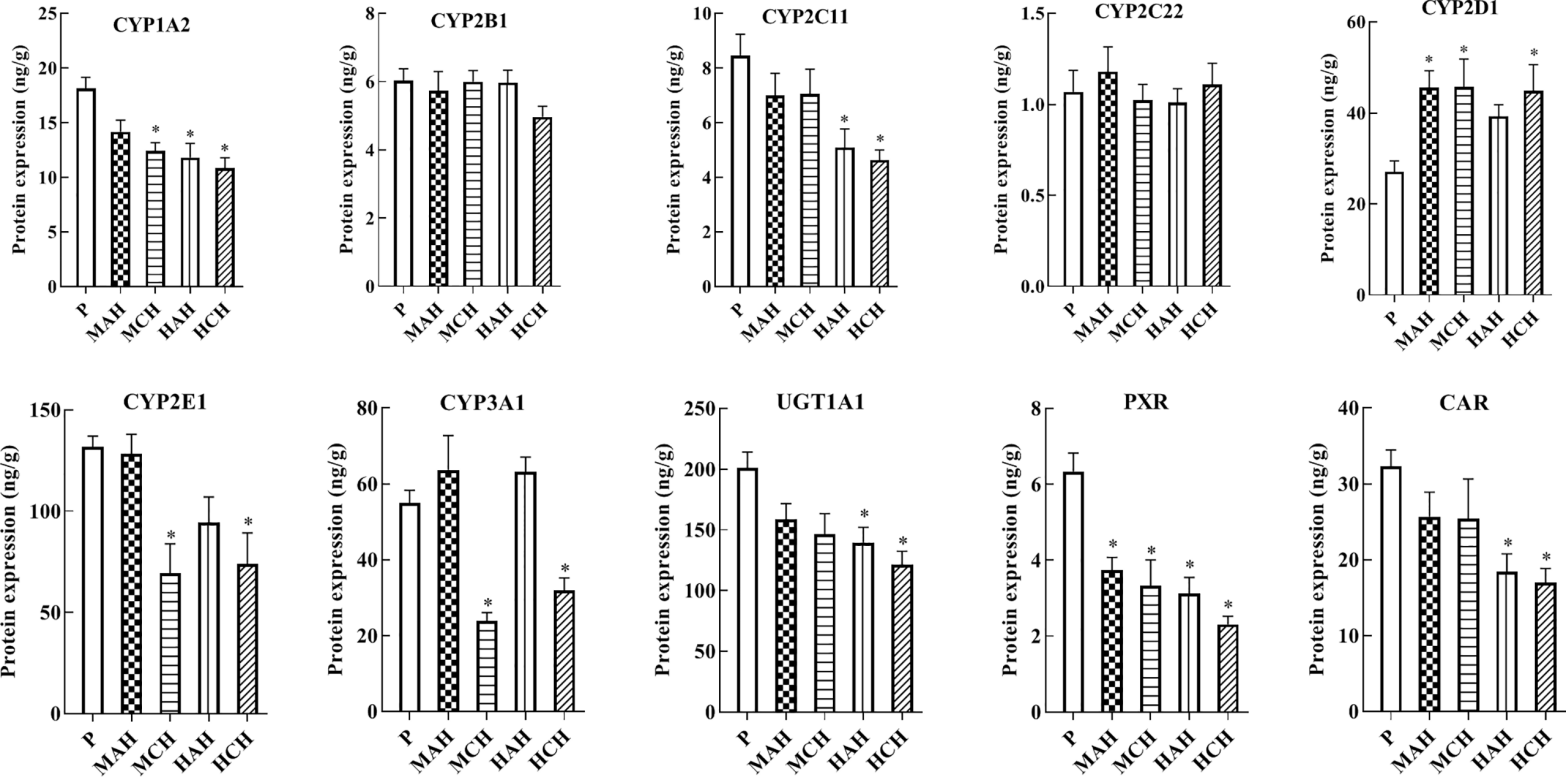
Protein expression of CYP1A2, CYP2B1, CYP2C11, CYP2C22, CYP2D1, CYP2E1, CYP3A1, UGT1A1, PXR, and CAR in rats. P: plain group (altitude: 390 m, PaO_2_: 20 kPa), MAH: acute moderate-altitude hypoxia group (altitude: 2,800 m, PaO_2_: 15.1 kPa; Rats were exposed to moderate altitude for 24 h), MCH: chronic moderate-altitude hypoxia group (altitude: 2,800 m, PaO_2_: 15.1 kPa; Rats were exposed to moderate altitude for 30 days), HAH: acute high-altitude hypoxia group (altitude: 4,300 m, PaO_2_: 12.4 kPa; Rats were exposed to high altitude for 24 h), HCH: chronic high-altitude hypoxia group (altitude: 4,300 m, PaO_2_: 12.4 kPa; Rats were exposed to high altitude for 30 days). The data were analyzed using a one-way analysis of variance (ANOVA), and the differences between the means of two groups were compared using LSD tests. ^*^ indicates the significance of experimental groups, compared with that of the control. Values are expressed as Mean ± SEM (n = 10). Levels are considered significant at ^*^ *p <* 0.05.

To reveal the molecular mechanisms underlying changes in drug metabolism during hypoxia, drug metabolic enzymes and nuclear receptors were examined by ELISA. After 24 h and 30-day treatment with high-altitude hypoxia, the protein expression of CYP1A2, CYP2C11, CYP3A1, CYP2E1, UGT1A1, PXR, and CAR was markedly decreased. However, the protein expression of CYP2D1 was markedly increased. The protein expression of CYP2B1 and CYP2C22 was not markedly changed (**Figure 2** and **Table S1**).

### mRNA Expression of CYP1A2, CYP2B1, CYP2C11, CYP2C22, CYP2D1, CYP2E1, CYP3A1, UGT1A1, PXR, and CAR in Rats

**Figure 3:**
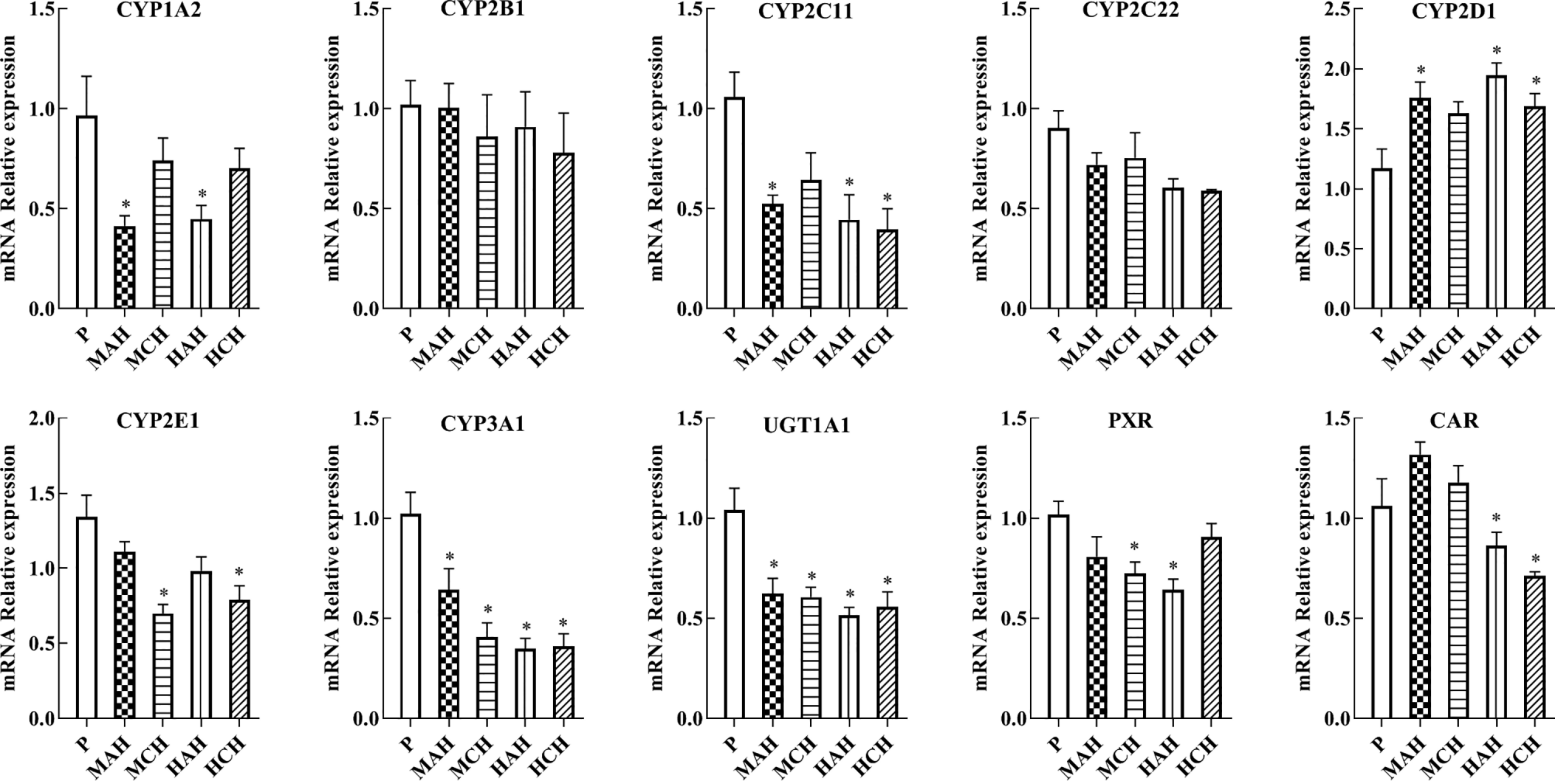
mRNA expression of CYP1A2, CYP2B1, CYP2C11, CYP2C22, CYP2D1, CYP2E1, CYP3A1, UGT1A1, PXR, and CAR in rats. P: plain group (altitude: 390 m, PaO_2_: 20 kPa), MAH: acute moderate-altitude hypoxia group (altitude: 2,800 m, PaO_2_: 15.1 kPa; Rats were exposed to moderate altitude for 24 h), MCH: chronic moderate-altitude hypoxia group (altitude: 2,800 m, PaO_2_: 15.1 kPa; Rats were exposed to moderate altitude for 30 days), HAH: acute high-altitude hypoxia group (altitude: 4,300 m, PaO_2_: 12.4 kPa; Rats were exposed to high altitude for 24 h), HCH: chronic high-altitude hypoxia group (altitude: 4300 m, PaO_2_: 12.4 kPa; Rats were exposed to high altitude for 30 days). The data were analyzed using a one-way analysis of variance (ANOVA), and the differences between the means of two groups were compared using LSD tests. ^*^ indicates the significance of experimental groups, compared with that of the control. Values are expressed as Mean ± SEM (n = 10). Levels are considered significant at ^*^ *p <* 0.05.

To confirm the downregulation of gene expression encoding the proteins of interest, mRNA expressions of drug metabolic enzymes and nuclear receptors were quantified by qPCR after 24 h and 30 days of treatment with high-altitude hypoxia. Changes in gene expression were similar to the corresponding changes in protein expression. The mRNA expression of CYP1A2, CYP2C11, CYP2E1, CYP3A1, PXR, and CAR were significantly decreased in hypoxia. The mRNA expression of CYP2D1 was increased in hypoxia. There was no significant difference in the mRNA expression of CYP2B1 and CYP2C22 between groups (**Figure 3** and **Table S1**).

### Protein Expression of CYP1A2, CYP2B6, CYP2C9, CYP3A4, UGT1A1, PXR, and CAR in HepG2 Cells

**Figure 4:**
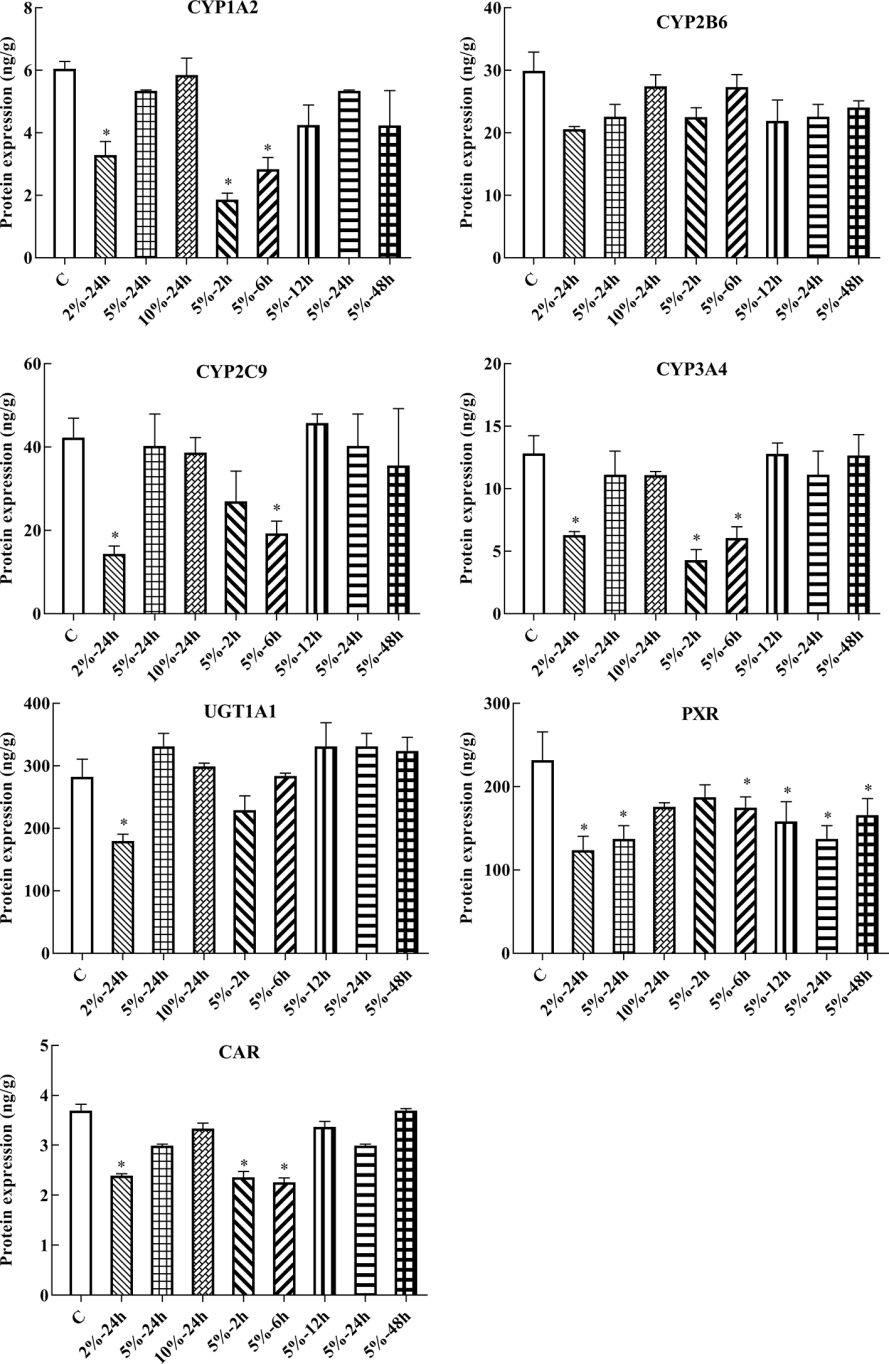
Protein expression of CYP1A2, CYP2B6, CYP2C9, CYP3A4, UGT1A1, PXR, and CAR in HepG2 cells. C, control group, HepG2 cells were cultured in a normoxic humidified incubator. HepG2 cells were exposure to 2% O_2_ for 24 h (2%-24 h), 5% O_2_ for 24 h (5%-24 h), 10% O_2_ for 24 h (10%-24 h), 5% O_2_ for 2 h (5%-2 h), 5% O_2_ for 6 h (5%-6 h), 5% O_2_ for 12 h (5%-12 h), 5% O_2_ for 24 h (5%-24 h), and 5% O_2_ for 48 h (5%-48 h). The data were analyzed using a one-way analysis of variance (ANOVA), and the differences between the means of two groups were compared using LSD tests. ^*^ indicates the significance of experimental groups, compared with that of the control. Values are expressed as Mean ± SEM (n = 3). Levels are considered significant at ^*^ *p <* 0.05.

In this experiment, HepG2 cells were used to assess changes in drug metabolic enzymes and nuclear receptors in hypoxia, and to study whether the changes in rats correspond to changes in HepG2 cells. After 2, 6, 12, 24, and 48 h treatment with 5% O_2_, and 24 h treatment with 2, 5, and 10% O_2_, protein expression was assessed by ELISA. The protein expression of CYP1A2, CYP2C9, CYP3A4, UGT1A1, PXR, and CAR were significantly decreased under hypoxic conditions. There was not significant difference in the protein expression of CYP2B6 in hypoxia (**Figures 4**, **6A** and **Table S2**).

### mRNA Expression of CYP1A2, CYP2B6, CYP2C9, CYP3A4, UGT1A1, PXR, and CAR in HepG2 Cells

**Figure 5:**
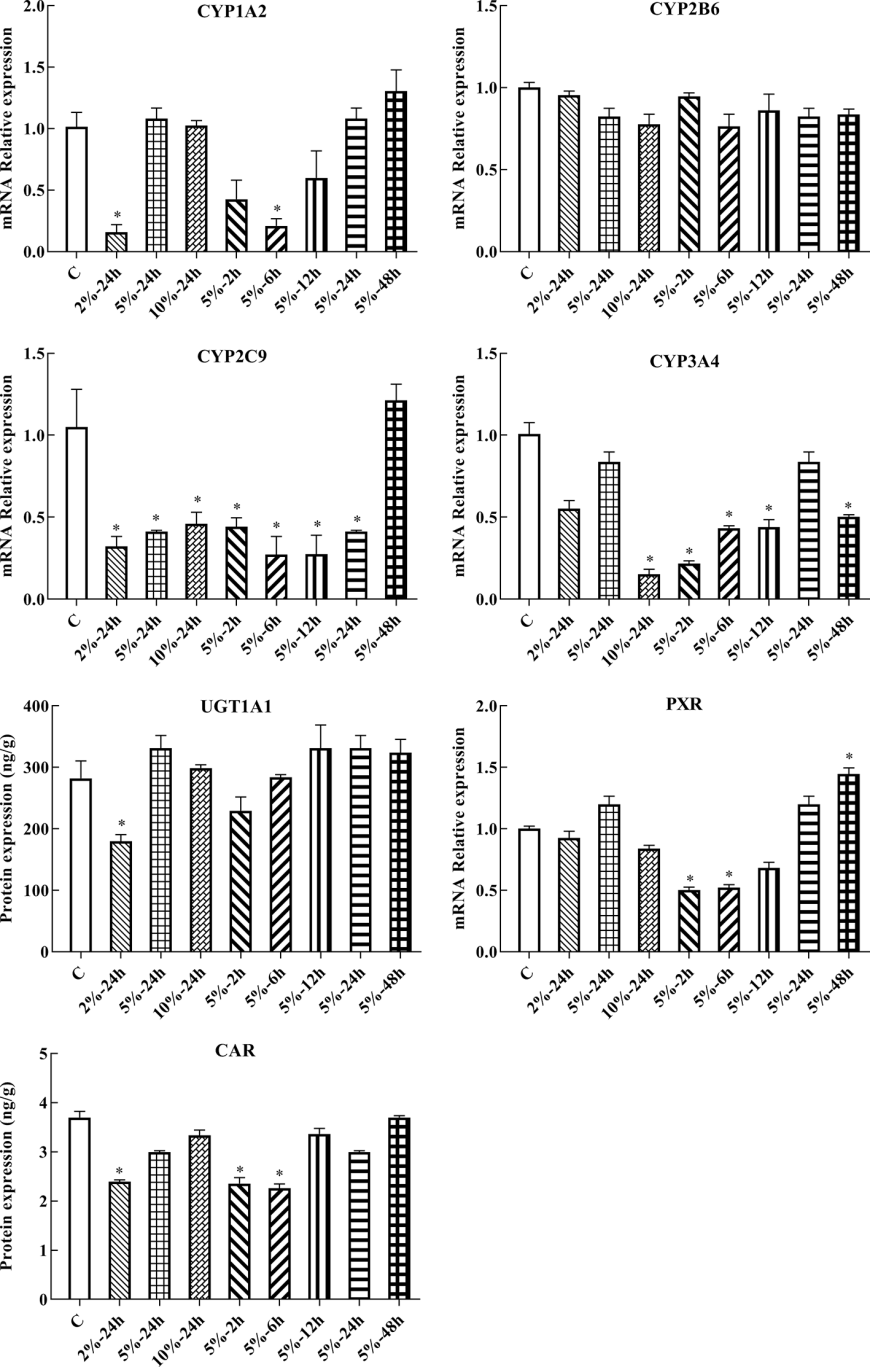
mRNA expression of Cyp1A2, Cyp2B6, Cyp2C9, Cyp3A4, Ugt1A1, Pxr, and Car in HepG2 cells. C: control group, HepG2 cells were cultured in a normoxic humidified incubator. HepG2 cells were exposure to 2% O_2_ for 24 h (2%-24 h), 5% O_2_ for 24 h (5%-24 h), 10% O_2_ for 24 h (10%-24 h), 5% O_2_ for 2 h (5%-2 h), 5% O_2_ for 6 h (5%-6 h), 5% O_2_ for 12 h (5%-12 h), 5% O_2_ for 24 h (5%-24 h), and 5% O_2_ for 48 h (5%-48 h). The data were analyzed using a one-way analysis of variance (ANOVA), and the differences between the means of two groups were compared using LSD tests. ^*^ indicates the significance of experimental groups, compared with that of the control. Values are expressed as Mean ± SEM (n = 3). Levels are considered significant at ^*^ *p <* 0.05.

**Figure 6:**
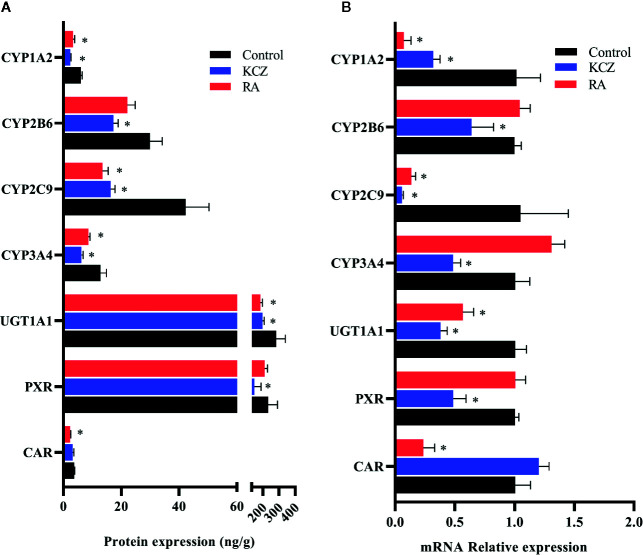
Expression of CYP1A2, CYP2B6, CYP2C9, CYP3A4, UGT1A1, PXR, and CAR in HepG2 cells following treatment with KCZ and RA. **(A)** The protein expression levels of CYP450 isoforms, UGT1A1, PXR, and CAR. **(B)** The mRNA expression levels of CYP450 isoforms, UGT1A1, PXR, and CAR. KCZ (Ketoconazole): PXR inhibitors; RA (Retinoic acid): CAR inhibitors. The data were analyzed using a one-way analysis of variance (ANOVA), and the differences between the means of two groups were compared using LSD tests. ^*^ indicates the significance of experimental groups, compared with that of the control. Values were expressed as Mean ± SEM (n = 3). Levels are considered significant at ^*^ *p <* 0.05.

Following 2, 6, 12, 24, and 48 h treatment with 5% O_2_, and 24 h treatment with 2, 5, and 10% O_2_, the mRNA expression of genes of interest was assessed by qPCR. Gene expression patterns in HepG2 cells were similar to the corresponding patterns observed in rats. The mRNA expression of CYP1A2, CYP2C9, CYP3A4, UGT1A1, PXR, and CAR were significantly decreased under hypoxic conditions. There was not significant difference in the mRNA expression of CYP2B6 in hypoxia (**Figures 5**, **6B** and **Table S2**).

### Promoter Activity of CYP1A2, CYP2C9, CYP2E1, CYP3A4, and UGT1A1 Under Hypoxic Conditions

**Figure 7:**
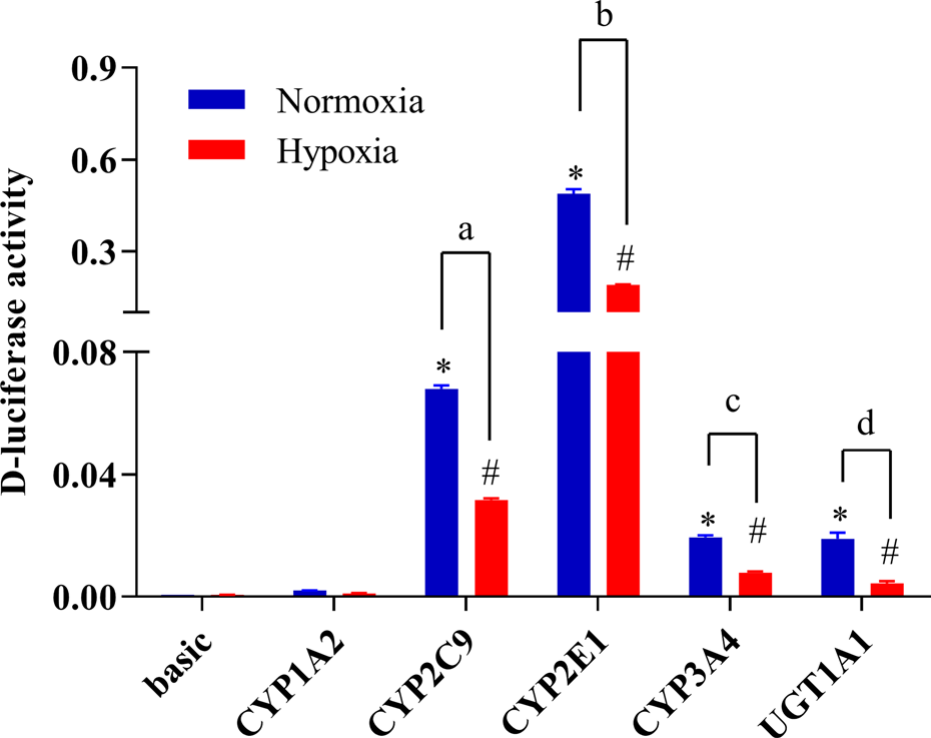
The promoter activity of CYP1A2, CYP2C9, CYP2E1, CYP3A4, and UGT1A1 under hypoxic conditions. The HepG2 cells were transfected with pGL3-CYP1A2, pGL3-CYP2C9, pGL3-CYP2E1, pGL3-CYP3A4, pGL3-UGT1A1, or empty vector. The HepG2 cells treatment with hypoxia (2% O_2_) or normoxia (21% O_2_) for 24 h after transfection. The data were analyzed using a one-way analysis of variance (ANOVA), and the differences between the means of two groups were compared using LSD tests. ^*,#,a,b,c,d^ indicates the significance of each of the experimental groups compared with that of the control. ^*^ *p <* 0.05 *vs.* normoxia basic; ^#^ *p <* 0.05 *vs.* hypoxia basic; ^a^ *p <* 0.05 *vs.* normoxia CYP2C9; ^b^ *p <* 0.05 *vs.* normoxia CYP2E1; ^c^ *p <* 0.05 *vs.* normoxia CYP3A4; ^d^ *p <* 0.05 *vs.* normoxia UGT1A1. Values were expressed as Mean ± SEM (n = 3). Levels are considered significant at ^*^ *p <* 0.05, ^#^ *p <* 0.05, ^a^ *p <* 0.05, ^b^ *p <* 0.05, ^c^ *p <* 0.05, and ^d^ *p <* 0.05.

To study changes in the expression of drug metabolic enzymes in hypoxia, the promoter activity of CYP1A2, CYP2C9, CYP2E1, CYP3A4, and UGT1A1 were examined using a luciferase reporter system in HepG2 cells. Transcriptional activity of CYP1A2, CYP2C9, CYP2E1, CYP3A4, and UGT1A1 were assessed using the pGL3-CYP1A2, pGL3-CYP2C9, pGL3-CYP2E1, pGL3-CYP3A4, and pGL3-UGT1A1 vectors, respectively. The promoter activities of CYP2C9, CYP2E1, CYP3A4, and UGT1A1 were significantly decreased under hypoxia. Compared with the normoxia group, 2% O_2_ decreased the promoter activity of CYP2C9, CYP2E1, CYP3A4, and UGT1A1 by 53.3, 61.3, 59.3, and 76.8%, respectively (**Figure 7**).

### The mRNA Expression of CYP1A2, CYP2C9, CYP2E1, CYP3A4, and UGT1A1 in PXR- and CAR-Silenced HepG2 Cells

**Figure 8:**
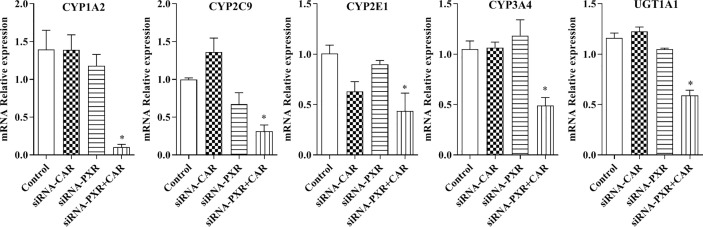
mRNA expression of CYP1A2, CYP2C9, CYP2E1, CYP3A4, and UGT1A1 after treatment with siRNA-PXR and siRNA-CAR. Following transfection with the siRNA-PXR and siRNA-CAR vectors, HepG2 cells were incubated for 24 h under normoxia. The data were analyzed using a one-way analysis of variance (ANOVA), and the differences between the means of two groups were compared using LSD tests. ^*^ indicates the significance of experimental groups, compared with that of the control. Values were expressed as Mean ± SEM (n = 3). Levels are considered significant at ^*^ *p <* 0.05.

Following the transfection of HepG2 cells with empty vector, siRNA-PXR and siRNA-CAR, and CYP1A2, CYP2C9, CYP2E1, CYP3A4, and UGT1A1 mRNA expression were assessed by qPCR. We investigated the possible roles of PXR and CAR in the regulation of the CYP1A2, CYP2C9, CYP2E1, CYP3A4, and UGT1A1 genes using PXR- and CAR-silenced HepG2 cells. The mRNA expression of CYP1A2, CYP2C9, CYP2E1, CYP3A4, and UGT1A1 were decreased 90.0, 92.9, 69.0, 56.4, 53.3, and 49.1%, respectively, in HepG2 cells transfected with siRNA-PXR+CAR. The mRNA expression of CYP1A2, CYP2C9, CYP2E1, CYP3A4, and UGT1A1 was not significantly changed in HepG2 cells transfected with siRNA-PXR or siRNA-CAR (**Figure 8**).

### The Promoter Activity of CYP1A2, CYP2C9, CYP2E1, CYP3A4, and UGT1A1 Following Treatment With KCZ and RA

**Figure 9:**
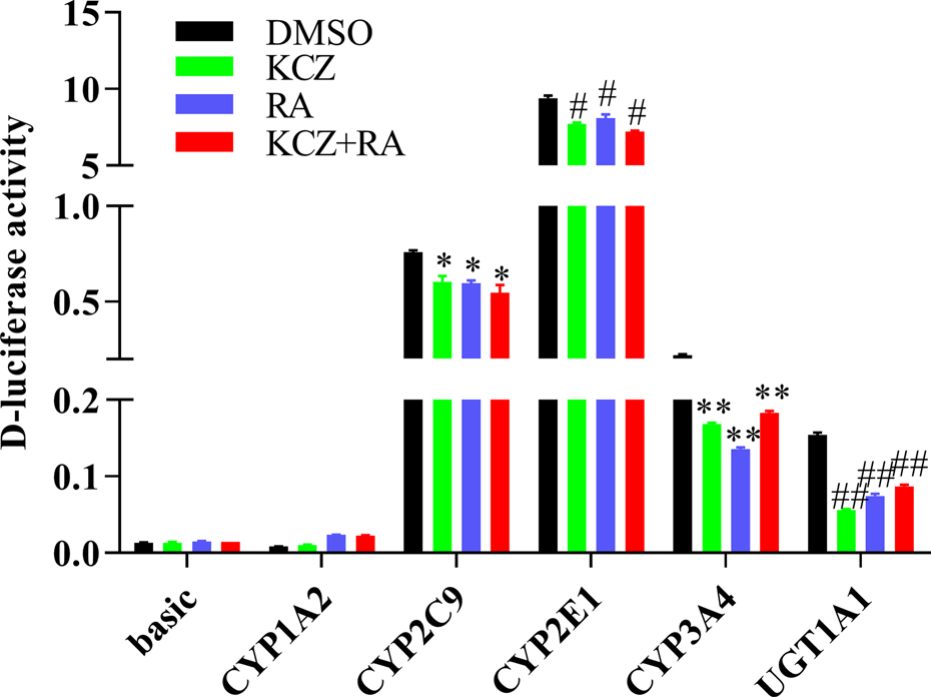
The promoter activity of CYP1A2, CYP2C9, CYP2E1, CYP3A4, and UGT1A1 following treatment with KCZ and RA. HepG2 cells were transfected with pGL3-CYP1A2, pGL3-CYP2C9, pGL3-CYP2E1, pGL3-CYP3A4, pGL3-UGT1A1, or empty vector. The HepG2 cells treatment with KCZ or RA after transfection in normoxia. KCZ (Ketoconazole): PXR inhibitors; RA (Retinoic acid): CAR inhibitors. The data were analyzed using a one-way analysis of variance (ANOVA), and the differences between the means of two groups were compared using LSD tests. ^*,#,**,##^ indicates the significance of each of the experimental groups compared with that of the control. ^*^ *p <* 0.05 *vs.* DMSO-CYP2C9; ^#^ *p <* 0.05 *vs.* DMSO-CYP2E1; ^**^ *p <* 0.05 *vs.* DMSO-CYP3A4; ^##^ *p <* 0.05 *vs.* DMSO-UGT1A1. Values were expressed as Mean ± SEM (n = 3). Levels are considered significant at ^*^ *p <* 0.05, ^#^ *p <* 0.05, ^**^ *p <* 0.05, ^##^ *p <* 0.05.

To determine whether the effects of KCZ and RA on the expression of CYP1A2, CYP2C9, CYP2E1, CYP3A4, and UGT1A1 were similar to the corresponding changes in PXR- and CAR-silenced HepG2 cells experiment. After treatment with KCZ, RA, and KCZ+RA, the promoter activities of CYP2C9, CYP2E1, CYP3A4, and UGT1A1 were decreased. The promoter activities of CYP2C9 decreased by 20.7, 21.5, and 28.1% in the KCZ, RA, and KCZ+RA groups, respectively, while that of CYP2E1 decreased by 18.1, 14.0, and 23.2% in the KCZ, RA, and KCZ+RA groups, respectively. In addition, that of CYP3A4 decreased by 23.4, 38.5, and 16.8% in the KCZ, RA, and KCZ+RA groups, respectively, and that of UGT1A1 decreased by 63.5, 51.8, and 43.8% in the KCZ, RA, and KCZ+RA groups, respectively, as compared to the DMSO group (**Figure 9**). These results are consistent with those obtained in the PXR- and CAR-silencing experiments.

### The mRNA Expression of CYP1A2, CYP2C9, CYP2E1, CYP3A4, and UGT1A1 Following Treatment With RIF and CITCO

**Figure 10:**
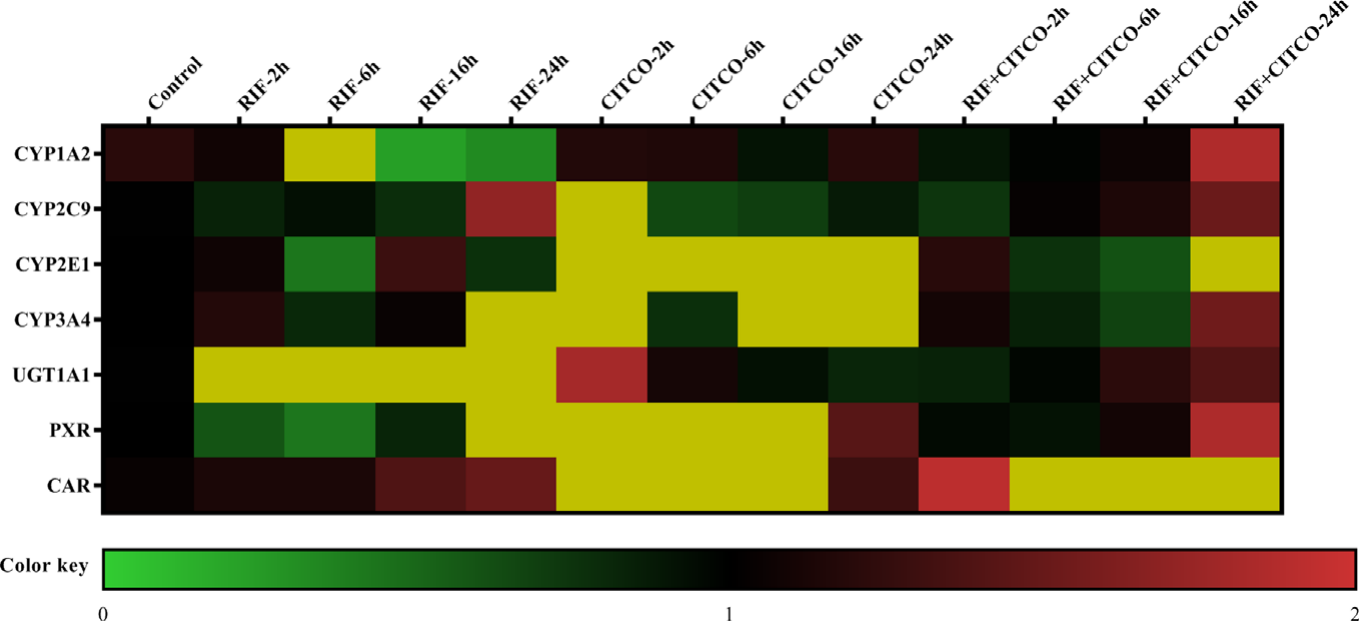
mRNA expression of CYP1A2, CYP2C9, CYP2E1, CYP3A4, and UGT1A1 following treatment with Rif and CITCO. HepG2 cells treatment with Rif or COTCO in normoxia, and the mRNA expression of CYP1A2, CYP2C9, CYP2E1, CYP3A4, and UGT1A1 were detected. The mRNA expression levels of CYP450 and UGT1A1 genes are shown using the indicated pseudocolor scale from 0 (green) to 2 (red) relative to the value for HepG2 cells in the control group. The color scale represents the relative mRNA expression levels, with green indicating down-regulated genes, red indicating up-regulated genes, dark yellow indicating over 2-fold up-regulated genes, and black indicating unchanged genes. Rif (rifampicin): PXR agonist, CITCO (6-(4-chlorophenyl)-imidazo (2,1-b) thiazole-5-carbaldehyde): CAR agonist.

To further study the regulation of CYP450 and UGT1A1 transcription by PXR and CAR, HepG2 cells were treated with Rif (10 μmol/L) and CITCO (10 μmol/L) for 2, 6, 16, and 24 h. Following treatment with Rif, the mRNA expression of PXR, CYP2C9, CYP3A4, and UGT1A1 was increased by 108.0, 70.0, 286.0, and 425.4%, respectively, in the RIF-24 h group. The mRNA expression of CYP1A2 increased by 160.0% in the RIF-6 h group. The mRNA expression of CYP2E1 increased by 28.0% in the RIF-16 h group. Rif did not induce CAR expression. Following treatment with CITCO for 2 h, the mRNA expression of CAR, CYP1A2, CYP2C9, CYP2E1, CYP3A4, and UGT1A1 increased by 202.0, 787.4, 1,018.8, 80.0, 263.4, and 808.9%, respectively. CITCO did not induce PXR expression. The mRNA expression of PXR, CAR, CYP1A2, CYP2C9, CYP2E1, CYP3A4, and UGT1A1 increased significantly by 85.0, 52.0, 106.0, 51.0, 39.0, 84.0, and 231.68%, respectively, in the RIF+CITCO-24 h group (**Figure 10**).

### Promoter Activity of CYP1A2, CYP2C9, CYP2E1, CYP3A4, and UGT1A1 Following Treatment With Rif and CITCO Under Different Oxygen Concentrations

**Figure 11:**
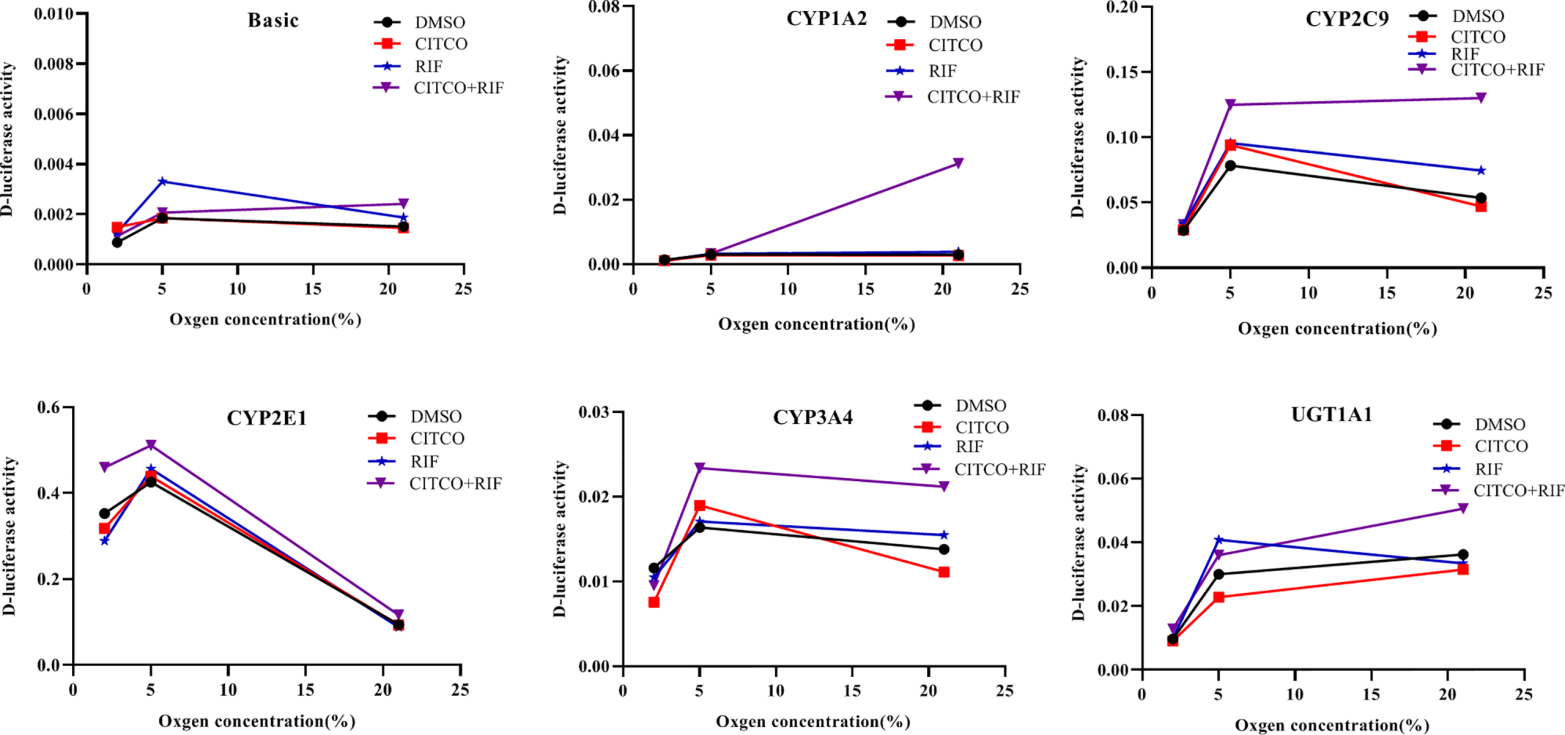
The promoter activity of CYP1A2, CYP2C9, CYP2E1, CYP3A4, and UGT1A1 following agonist treatment under different oxygen concentrations. HepG2 cells were treated with RIF or CITCO under 2, 5, and 21% O_2_, after which the drug-containing medium was removed, and the D-luciferase activity of CYP450 and UGT1A1 were measured. n = 3 independent experiments in triplicate; a representative graph is shown. Rif (rifampicin): PXR agonist, CITCO (6- (4-chlorophenyl)-imidazo (2,1-b) thiazole-5-carbaldehyde): CAR agonist.

To investigate whether the altered expression of CYP1A2, CYP2C9, CYP2E1, CYP3A4, and UGT1A1 under hypoxia is dependent on PXR and CAR, gene promoter activities were determined in HepG2 cells following treatment with Rif and CITCO under different oxygen concentrations using a luciferase reporter system. The effects of agonists on CYP1A2, CYP2C9, CYP2E1, CYP3A4, and UGT1A1 promoter activity under different oxygen concentrations revealed that the promoter activity of CYP2E1 was decreased significantly by 9.8 and 18.2% in the CITCO and RIF groups at 2% O_2_, respectively. However, the promoter activity of CYP2E1 increased significantly by 30.3% in the CITCO+RIF group. At 5% O_2_, the promoter activity of CYP2E1 increased by 7.3 and 20.0% in the RIF and RIF+CITCO groups, respectively. The promoter activity of CYP2C9 increased by 59.2% in the RIF+CITCO group. Under normoxic conditions, the promoter activities of UGT1A1, CYP1A2, CYP3A4, CYP2C9, and CYP2E1 were increased by 39.8, 941.0, 53.5, 142.5, and 24.0%, respectively, in the RIF+CITCO group. The promoter activity of CYP2C9 increased by 39.1% in the RIF group (**Figure 11**).

## Discussion

We examined the physiological and gene transcriptional changes in rats after acute or chronic high-altitude exposure, the contents of RBC, HGB, and BIL were significantly increased in high-altitude hypoxia. The values for S_c_O_2_ in the MAH, MCH, HAH, and HCH groups were significantly decreased compared to the P group. In the current study, we found that hypoxia affected expression of drug metabolism enzymes and nuclear receptors, and determined that PXR and CAR were the mechanisms for regulation of CYP450 and UGT1A1 in hypoxia. In our previous study, we found that physiologic and blood parameters affect the pharmacokinetics of sulfamethoxazole. Sulfamethoxazole is highly bound to proteins primarily to albumin; bilirubin also binds to albumin and might reduce the protein binding of sulfamethoxazole. We found that the binding of sulfamethoxazole to red blood cells increased after exposure hypoxia (Li et al., 2012). Physiologic and blood parameters are also regarded as important criteria in the evaluation of an animal model. The CYP450 enzymes are a superfamily with related structure and function of isozymes involved in the endogenous metabolism and detoxification of exogenous substances (Li et al., 2017). Humans and rats are both mammals, however, and CYP450 subtypes are unique to each species (Lu et al., 2016). CYP1A2, CYP2B1, CYP2C11, CYP2C22, CYP2D1, CYP2E1, CYP3A1, UGT1A1, PXR, and CAR from rats were selected for the present study. CYP1A2, CYP2B6, CYP2C9, CYP3A4, UGT1A1, PXR, and CAR were investigated in human HepG2 cells.

Due to the extreme environment of the plateau, in addition to poor experimental conditions, many studies simulate high-altitude environments in a decompression chamber (Souich and Fradette, 2011; Gola et al., 2013; Gola et al., 2016). However, these two settings are different with regard to the study of drug metabolism enzymes, and it is difficult to reproduce the high-altitude environment in a low-altitude laboratory. The present study was performed in Gonghe county (altitude: 2,800 m, PaO_2_: 15.1 kPa) and Huashixia town (altitude: 4,300 m, PaO_2_: 12.4 kPa), both of which are located in the Northwestern Qinghai Province of China. We found that both the protein and mRNA expression of PXR, CAR, CYP1A2, CYP2C11, CYP2E1, CYP3A1, and UGT1A1 were decreased, while those of CYP2D1 increased in the high-altitude environment. However, there was no significant change in the expression of CYP2B1 and CYP2C22 in the high-altitude environment; this is similar to a trend observed in our previous study (Li et al., 2014). Hao and Shefali exposed rats to hypobaric hypoxia for 6 and 24 h at a simulated altitude of 7,620 m and reported that the activity of UGT was not significantly changed (Hao et al., 2013; Shefali et al., 2013). This can be explained in various ways; however, the key factors are the different altitude levels and hypoxic environment. We found that hypoxia caused significant decreases in the activity of CYP450 in rats in previous study (Li et al., 2014). Due to the translational *in vivo* effect of these results in terms of drug metabolism activity and pharmacokinetics cannot be extrapolated, there are some limitations in activity assessment of drug metabolizing enzymes under hypoxic conditions. The high-altitude environment is characterized by increased solar radiation, decreased ambient oxygen tension, extreme diurnal ranges in temperature, arid climate, and poor soil quality; of these, hypoxia is the main factor with the potential to affect human life and activity (Eide and Asplund, 2012). In addition to hypoxia, whether other factors that radiation, temperature, humidity, different altitudes, the duration of hypoxia, and the hypoxic environment are highly important variables, affecting CYP450 and UGT1A1 under hypoxia environment needs further investigation.

Few studies have investigated changes in the protein and mRNA expression of CYP1A2, CYP2B6, CYP2C9, CYP3A4, UGT1A1, PXR, and CAR *in vitro* under different oxygen concentrations. The data presented herein suggest that the protein and mRNA expression of PXR, CAR, CYP1A2, CYP2C9, CYP3A4, and UGT1A1 were significantly decreased following exposure to 2% O_2_ for 24 h. With increasing oxygen concentration and prolonged exposure, there was little change in the expression of PXR, CAR, CYP1A2, CYP2C9, CYP3A4, and UGT1A1. The protein and mRNA expression of CYP2B6 were not significantly changed in HepG2 cells treated with different oxygen concentrations for different times. However, Jürgens et al. found that hypoxia did not affect the activity of CYP1A2, which led to a decrease in the activity of CYP3A4 and CYP2D6 (Jürgens et al., 2002). These divergent results may be due to the different research methods and experimental subject. To study the effects of PXR and CAR on the transcriptional activity of CYP1A2, CYP2B6, CYP2C9, CYP3A4, and UGT1A1, HepG2 cells were treated with inhibitors of PXR (KCZ) and CAR (RA). Both CYP2B6 protein and mRNA expression were not significantly altered in HepG2 in hypoxia and after treated with RA, which might be that RA exert complex effects on CYP450 gene expression (Chen et al., 2010), and there are many factors which are related to the regulation of the CYP2B6. Given our results, we suggest that CYP2B6 and CYP3A4 transcription were mediated by PXR, while that of CYP1A2, CYP2C9, and UGT1A1 were mediated by PXR and CAR in normoxia.

Based on changes in the expression of CYP1A2, CYP2B6, CYP2C9, CYP3A4, UGT1A1, PXR, and CAR under different oxygen concentrations, 2% O_2_ was selected for the transient transfection experiment. The promoter activities of CYP2C9, CYP2E1, CYP3A4, and UGT1A1 were significantly decreased under hypoxic conditions. However, the activity of CYP1A2 was not significantly changed. It is possible that hypoxia inhibited the mRNA expression and promoter activity of CYP2C9, CYP2E1, CYP3A4, and UGT1A1. However, the results obtained for mRNA expression differed from those obtained for promoter activity in CYP1A2. This may be explained by the liver-enriched transcription factors (LETFs), which play crucial roles in the control of liver-specific gene expression, including hepatocyte nuclear factor 1 (HNF1), HNF3, HNF4, HNF6, CCAAT/enhancer-binding protein (C/EBP), and D-site binding protein (DBP) (Schrem and Klempnauer, 2002; Akiyama and Gonzales, 2003). Borlak et al. (2015) found that the gene expression of C/EBP-α increased in cultured hepatocytes, and CYP1A2 gene expression increased *in vitro* (Borlak et al., 2015).

Following treatment with si-PXR/CAR, the mRNA expression of CYP1A2, CYP2C9, CYP2E1, CYP3A4, and UGT1A1 were decreased. To further study the effects of PXR and CAR on the transcriptional activity of CYP1A2, CYP2E1, CYP2C9, CYP3A4, and UGT1A1, HepG2 cells were treated with KCZ and RA and then the promoter activities of CYP2C9, CYP2E1, CYP3A4, and UGT1A1 were found to be decreased. However, the promoter activity of CYP1A2 was not significantly changed. This was consistent with changes in the expression of CYP1A2 under hypoxia. Chang et al. (2017) reported that ursolic acid (UA) could inhibit the effects of PXR and CAR, and reduce the expression and function of CYP3A4, thus confirming the effects of PXR and CAR on the transcriptional regulation of CYP3A4 (Chang et al., 2017). To further support the role of PXR and CAR in the transcriptional regulation of CYP450 under hypoxia, we found that the mRNA expression of CYP1A2, CYP2C9, CYP2E1, CYP3A4, and UGT1A1 were increased following treatment with PXR and CAR agonists in normoxia. In the current study, we found that the promoter activities of CYP1A2, CYP2C9, CYP3A4, and UGT1A1 were not significantly changed under 2% O_2_, and those of CYP1A2, CYP3A4, and UGT1A1 were not significantly changed under 5% O_2_ following treatment with PXR and CAR agonists. When the oxygen concentration was increased to 21% (normoxia), the promoter activities of CYP1A2, CYP2C9, CYP2E1, CYP3A4, and UGT1A1 were increased following treatment with PXR and CAR agonists. It is possible that hypoxia antagonized the activating effects of PXR and CAR agonists. The II phase drug-metabolizing enzyme UGT1A1 is closely associated with adverse drug reaction (Schulz et al., 2009). We found that the mRNA expression of UGT1A1 was decreased in hypoxia and demonstrated the effects of PXR and CAR on the transcriptional activity of UGT1A1. Vyhlidal et al. and Kato et al. also found that UGT1A1 gene expression was regulated by PXR and CAR (Vyhlidal et al., 2004; Kato et al., 2016). In the current study, we found that hypoxia affected expression of PXR, CAR, CYP1A2, CYP2C9, CYP2E1, CYP3A4, and UGT1A1. These data are consistent with other studies showing that CYP450, UGT1A1, PXR, and CAR were decreased in hypoxia (Chen et al., 2004; Ratajewski et al., 2011; Hu et al., 2011; Park et al., 2012), and thus, taken together, this evidence points to PXR and CAR as the possible mechanism for regulation of CYP1A2, CYP2C9, CYP2E1, CYP3A4, and UGT1A1 in hypoxia.

This study found an important role of nuclear receptors PXR and CAR in regulation of CYP450 and UGT1A1 in hypoxia. Other studies shown that hypoxia significantly increased the expression of AhR (Aryl hydrocarbon receptor) who is the main transcription factor affecting CYP450 1 enzymes (Soo and Joon, 2020; Nuno et al., 2020). Whether AhR affects CYP450 and UGT1A1 under hypoxia environment needs further evaluation.

To our knowledge, this is the first report to identify a key role for PXR and CAR in drug metabolism enzymes at high-altitude hypoxia. The mechanisms underlying altered drug metabolism following exposure to hypoxic conditions have thus far remained unknown. The implication of our findings for research and future pharmacotherapy for high-altitude residents is substantial. Phase II drug-metabolizing enzymes require extensive research, and future research should include studies of nuclear receptor gene knockout rats under hypoxia.

## Data Availability Statement

The raw data supporting the conclusions of this article will be made available by the authors, without undue reservation, to any qualified researcher.

## Ethics Statement

The animal study was reviewed and approved by Animal Ethics Committee of the Qinghai University.

## Author Contributions

X-YL, NQ, and X-JW conceived and designed the research study. Y-BD, J-BZ, J-XY, and G-QL participated in the acquisition of samples and data. XB and Y-BD analyzed and interpreted the data. Y-BD, J-BZ, and X-YL wrote and revised the manuscript. All authors contributed to the article and approved the submitted version.

## Funding

This work was supported by the National Natural Science Foundation of China (No. 81760673 and 81660197) and the Natural Science Fund of Qinghai province, China (No. 2019-ZJ-918).

## Conflict of Interest

The authors declare that the research was conducted in the absence of any commercial or financial relationships that could be construed as a potential conflict of interest.

## Abbreviations

CYP450, cytochrome P450; UGT1A1, UDP-glucuronosyltransferase 1A1; PXR, pregnane X receptor; CAR, constitutive androstane receptor; AMS, acute mountain sickness; CMS, chronic mountain sickness; HPH, hypoxic pulmonary hypertension; HAPE, high-altitude pulmonary edema; CL, clearance; Vd, volume of distribution; T_1/2_, half-life; C_max_, peak concentration; PPAR, peroxisome proliferator-activated receptor; ELISA, enzyme-linked immunosorbent assay; RBC, red blood cells; WBC, white blood cells; HGB, hemoglobin; ScO_2_, blood oxygen saturation; ALB, albumin; BIL, bilirubin; KCZ, ketoconazole; RA, retinoic acid; RIF, rifampicin; CITCO, 6- (4-chlorophenyl)-imidazo (2,1-b) thiazole-5-carbaldehyde.
