# Supplementary material for: Regulation of High-Altitude Hypoxia on the Transcription of CYP450 and UGT1A1 Mediated by PXR and CAR
Source: Front Pharmacol. 2020 Sep 17;11:574176. doi: 10.3389/fphar.2020.574176 (PMC7527593; doi:10.3389/fphar.2020.574176)
Supplement: Supplementary file 1 [file DataSheet_1.zip › Supplementary Material.doc]

***Supplementary Material***

**Regulation of high-altitude hypoxia on the transcription of CYP450 and UGT1A1 mediated by PXR and CAR**

***Ya-bin Duan^1,2^***†***, Jun-bo Zhu^1,2^***†***, Jian-xin Yang^2^, Gui-qin Liu^3^, Xue Bai^1^, Ning Qu^4*^, Xue-jun Wang^5*^, Xiang-yang Li^2*^***

*^1^Research Center for High Altitude Medicine, Qinghai University Medical College, Xining, China, ^2^State Key Laboratory of Plateau Ecology and agriculture, Qinghai University, Xining, China, ^3^College of Eco-Environmental Engineering, Qinghai University, Xining, China, ^4^Department of Anesthesiology, Qinghai Hospital of Traditional Chinese Medicine, Xining, China, ^5^Department of Anesthesiology, Red Cross Hospital of Qinghai, Xining, China*

*** Correspondence:**

*Ning Qu*

*E-mail:* [*13997092887@163.com*](mailto:qnmzdr@163.com)

*Xue-jun Wang*

*E-mail:* [*wangxuejunhsz@163.com*](mailto:wangxuejunhsz@163.com)

*Xiang-yang Li*

*E-mail:* [*qhmclxy@163.com*](mailto:qhmclxy@163.com)

*† These authors share first authorship*

**Supplemental contains:**

**Figure S1. Amplification curves of SYBR green-based real-time qPCR assay (A). Melt curve (B). Melt Peak(C)**

**Figure S2. Total RNA tested by gel electrophoresis. M=2000 bp marker, Lanes 1-20= samples**

**Figure S3. SYBR green-based real-time qPCR products tested by gel electrophoresis. M=2000 bp marker**

**Figure S4. PCR identification of CYP1A2, CYP2C9, CYP3A4, CYP2E1, and UGT1A1 clonal colony. M=2000 bp marker.**

**Figure S5. pGL3-Basic, pGL3-Control, pRL-TK Vector map.**

**Figure S6. DNA sequencing reveals that genes were correctly placed in the plasmid.**

**Figure S7. Colony PCR of pGL3-CYP1A2, pGL3-CYP2C9, pGL3-CYP2E1, pGL3-CYP3A4 and pGL3-UGT1A1 (Maker DL 2000, from top to bottom: 2000, 1000, 750, 500, 250 and 100 bp)**

**Figure S8. Transfection efficiencies for cell lines following transfection with siRNA. Phase contrast (left panel) and fluorescence images (right panel) of cells 48 h after transfection. A and B: Control plasmid; C and D: siRNA-CY3.**

**Table S1. The change percentage of CYP450 expression under hypoxia in rats.**

**Table S2. The change percentage of CYP450 expression under hypoxia in HepG2 .**

**A**

**B**

**C**

**Figure S1. Amplification curves of SYBR green-based real-time qPCR assay (A). Melt curve (B). Melt Peak(C)**

20

19

18

17

16

15

14

13

12

11

10

9

8

7

6

5

4

3

2

1

M

**Figure S2. Total RNA tested by gel electrophoresis. M=2000 bp marker, Lanes 1-20= samples**

1901 bp (CYP1A2)

2051 bp (UGT1A1)

2001 bp (CYP3A4)

2051 bp (CYP2C9)

2100 bp (CYP2E1)

**Figure S3. SYBR green-based real-time qPCR products tested by gel electrophoresis. M=2000 bp marker.**

1901 bp (CYP1A2)

2100 bp (CYP2E1)

2051 bp (UGT1A1)

2051 bp (CYP2C9)

2001 bp (CYP3A4)

**Figure S4. PCR identification of CYP1A2, CYP2C9, CYP3A4, CYP2E1, and UGT1A1 clonal colony. M=2000 bp marker.**

**Figure S5. pGL3-Basic, pGL3-Control, pRL-TK Vector map.**

**CYP1A2：**

**CYP2B6：**

**CYP2C9：**

**CYP2E1:**

**CYP3A4：**

**UGT1A1：**

**Figure S6. DNA sequencing reveals that genes were correctly placed in the plasmid.**

**Figure S7. Colony PCR of pGL3-CYP1A2, pGL3-CYP2C9, pGL3-CYP2E1, pGL3-CYP3A4 and pGL3-UGT1A1 (Maker DL 2000, from top to bottom: 2000, 1000, 750, 500, 250 and 100 bp)**

A

B

C

D

**Figure S8. Transfection efficiencies for cell lines following transfection with siRNA. Phase contrast (left panel) and fluorescence images (right panel) of cells 48 h after transfection. A and B: Control plasmid; C and D: siRNA-CY3.**

**Table S1. The change percentage of CYP450 expression under hypoxia in rats.**

| Drug metabolizing enzymes | Effects |
| --- | --- |
| CYP1A2 | Protein expression decreased by 31.6%, 34.8%, and 40.3% in MCH, HAH, and HCH groups.  mRNA expression decreased by 57.7% and 53.6% in the MAH and HAH groups. |
| CYP2C11 | Protein expression decreased by 39.8% and 45.2% in the HAH and HCH groups.  mRNA expression decreased by 50.9%, 57.6% and 62.3% in the MAH, HAH, and HCH groups. |
| CYP2E1 | Protein expression decreased by 47.5% and 43.9% in the MCH and HCH groups.  mRNA expression decreased by 47.8% and 41.0% in the MCH and HCH groups |
| CYP3A1 | Protein expression decreased by 56.5% and 23.0% in the MCH and HCH groups.  mRNA expression decreased by 37.3%, 59.8%, 65.7% and 64.7% in the MAH, MCH, HAH, and HCH groups. |
| CYP2D1 | Protein expression increased by 68.0%, 68.9%, and 65.6% in the MAH, MCH, and HCH groups.  mRNA expression increased by 50.4%, 65.8%, and 44.4% in the MAH, HAH, and HCH groups. |
| UGT1A1 | Protein expression decreased by 30.6% and 39.7% in HAH and HCH groups.  mRNA expression decreased by 40.4%, 41.4%, 59.6%, and 46.2% in the MAH, MCH, HAH, and HCH groups. |
| PXR | Protein expression increased by 41.0%, 47.6%, 50.6%, and 63.6% in MAH, MCH, HAH, and HCH groups.  mRNA expression decreased by 29.4% and 37.3% in the MCH and HAH groups. |
| CAR | Protein expression increased by 43.1% and 47.4% in the HAH and HCH groups.  mRNA expression decreased by 8.9% and 33.0% in the HAH and HCH groups. |

P: plain group (altitude: 390 m, PaO_2_: 20 kPa), MAH: acute moderate altitude hypoxia group (altitude: 2800 m, PaO_2_: 15.1 kPa; Rats were exposed tomoderate altitude for 24 h), MCH: chronic moderate altitude hypoxia group (altitude: 2800 m, PaO_2_: 15.1 kPa; Rats were exposed to moderate altitude for 30 days), HAH: acute high-altitude hypoxia group (altitude: 4300 m, PaO_2_: 12.4 kPa; Rats were exposed to high-altitude for 24 h), HCH: chronic high-altitude hypoxia group (altitude: 4300 m, PaO_2_: 12.4 kPa; Rats were exposed to high-altitude for 30 days).

**Table S2. The change percentage of CYP450 expression under hypoxia in HepG2 .**

| Drug metabolizing enzymes | Effects |
| --- | --- |
| CYP1A2 | Protein expression decreased by 61.3%, 45.2%, 45.7%, 69.0%, and 53.1% in the KCZ, RA, 2%-24 h, 5%-2 h, and 5%-6 h groups.  mRNA expression decreased by93.1%, 84.3%, and 79.4% in the RA, 2%-24 h, and 5%-6 h groups. |
| CYP2C9 | Protein expression decreased by 61.4%, 68.0%, 66.2%, and 54.5% in the KCZ, RA, 2%-24 h, and 5%-6 h groups.  mRNA expression decreased by 94.3%, 86.7%, 74.4%, 61.0%, 56.2%, 58.1%, 74.3%, 73.3%, and 61.0% in the KCZ, RA, 2%-24 h, 5%-24 h, 10%-24 h, 5%-2 h, 5%-6 h, 5%-12 h, and 5%-24 h groups. |
| CYP3A4 | Protein expression decreased by 51.5%, 32.6%, 51.0%, 66.6%, and 52.7% in the KCZ, RA, 2%-24 h, 5%-2 h, and 5%-6 h groups.  mRNA expression decreased by 51.0%, 85.0%, 78.0%, 57.0%, 56.0%, and 50.0% in the KCZ, 10%-24 h, 5%-2 h, 5%-6 h, 5%-12 h, and 5%-48 h groups. |
| UGT1A1 | Protein expression decreased by 29.9%, 34.8%, and 36.3% in the KCZ, RA, and 2%-24 h groups.  mRNA expression decreased by 62.0%, 43.0%, 73.0%, 96.0%, and 44.0% in the KCZ, RA, 2%-24 h, 10%-24 h, and 5%-12 h groups. |
| PXR | Protein expression increased by 36.8%, 46.6%, 24.6%, 31.7%, 40.8%, and 28.5% in the KCZ, 2%-24 h, 5%-6 h, 5%-12 h, 5%-24 h, and 5%-48 h groups.  mRNA expression decreased by 51.0%, 50.0%, and 48.0% in the KCZ, 5%-2 h, and 5%-6 h groups. |
| CAR | Protein expression increased by 38.1%, 35.4%, 36.2% and, 38.9% in the RA, 2%-24 h, 5%-2 h, and 5%-6 h groups.  mRNA expression decreased by 76.0%, 55.0%, and 66.0% in the RA, 2%-24 h, and 10%-24 h groups. |

HepG2 cells were cultured in a normoxic humidified incubator. HepG2 cells were exposure to 2% O_2_ for 24 h (2%-24 h), 5% O_2_ for 24 h (5%-24 h), 10% O_2_ for 24 h (10%-24 h), 5% O_2_ for 2 h (5%-2 h), 5% O_2_ for 6 h (5%-6 h), 5% O_2_ for 12 h (5%-12 h), 5% O_2_ for 24 h (5%-24 h), and 5% O_2_ for 48 h (5%-48 h). KCZ (Ketoconazole): PXR inhibitors; RA (Retinoic acid): CAR inhibitors.
